# Fracture of the Cranium

**Published:** 1828-12

**Authors:** 


### FRACTURE OF THE CRANIUM.

### *Fracture of the Cranium, with extensive Depression; Operation.* (GLASGOW ROYAL INFIRMARY.)

DANIEL MACLEOD, aged eleven. About four o'clock P.M. while working inside the boiler of a steam-engine, one of the rivets used in fastening the parts of the boiler together was driven violently through by the blow of a hammer from one of the workmen, and struck his head. He was rendered insensible for some time, and lost a considerable quantity of blood from a wound of the scalp, an inch and a half in length, over the occipital angle of the right parietal bone. He was brought to the hospital two hours after the accident, and admitted under the care of Dr. MACLACHLAN.

On raising the detached portion of scalp, a fracture was brought to view, in diameter three-fourths of an inch, and depressed obliquely nearly half an inch. He laboured under some stupor, but was quite sensible. Pulse eighty-four, of moderate strength; motions of limbs unaffected.

Sumat statim Calomel ℈i.

Second and third day.—Was bled and purged, and went on favorably.

Fourth day.—Passed a good night. Countenance slightly flushed; pulse ninety; skin rather warm; tongue red and dry. Several stools.

On the sixth day he complained of pain in front of right ear, and on the seventh of pain in the vicinity of fracture; but in other respects was well. Pulse eighty. Towards the evening, however, he began to dose; and on the eighth is reported as having remained almost constantly in a torpid state. He vomited after taking breakfast, and could be brought to answer questions with difficulty. Respiration easy; pulse sixty-five; skin natural; pupils slightly dilated, and sluggish. A consultation advised immediate elevation of the depressed portion of bone.

A triangular incision was made in the scalp, and a piece of bone, nearly an inch square, was found depressed in the most prominent part of right parietal bone. This depressed piece of bone seemed to have yielded at the centre, as it was found divided into three or four parts, the fracture running from the centre to the circumference, and thus forming several triangular pieces, the acute angles of which were pressed directly down upon the brain. Two applications of the trephine were necessary before the pieces of bone could be raised. The internal table was found more depressed than the external, having wounded the dura mater at several points. The brain also had been wounded by the spicula, small portions of it being observed floating in the blood. The depressed brain and membranes rose to their natural situation: the boy immediately seemed more lively, his pupils became active, and his pulse rose from sixty-five to ninety-six. With the exception of a pretty smart feverish attack about a week afterwards, the boy did well; and exactly a month after the accident he was dismissed cured, the wound being healed and the pulsations under it scarcely perceptible. He has since come to shew himself in perfect health.

---

